# Unveiling the Broad-Spectrum Virucidal Potential of Purified Clinoptilolite-Tuff

**DOI:** 10.3390/microorganisms12081572

**Published:** 2024-08-01

**Authors:** Alisina Sarabi, Stéphane Nizet, Andreas Röhrich, Cornelius Tschegg

**Affiliations:** GLOCK Health, Science and Research GmbH, 2232 Deutsch-Wagram, Austria; alisina.sarabi@glock.at (A.S.); andreas.roehrich@glock.at (A.R.); cornelius.tschegg@glock.at (C.T.)

**Keywords:** zeolite, clinoptilolite, clinoptilolite-tuff, viruses, focus-forming assay

## Abstract

Due to its remarkable surface properties, natural clinoptilolite-tuff interacts with a variety of biochemical, pharmaceutical, chemical, and microbiological entities, including human viruses. In the present work, the virucidal activity of purified clinoptilolite-tuff (PCT) was investigated using a variety of viruses, differing in their structure and composition. Influenza A virus, Herpes Simplex virus, Rhinovirus, and Parvovirus were chosen to represent enveloped and non-enveloped viruses with RNA and DNA genomes. Beside human viruses, Canine Parvovirus and bacteriophages T4 and MS2 were used to represent animal and bacterial viruses, respectively. The virucidal activity of PCT was quantified by examining the residual viral activity on susceptible cell lines upon incubation with PCT. A wide range of antiviral efficiencies was observed, ranging from up to 99% for Herpes Simplex virus to no activity for Rhinovirus and both bacteriophages. This study reveals that the virucidal potential of PCT is not universal and depends on a complex set of factors including virus structure and medium composition. The environmental and medical implications of this research are discussed for uses such as wastewater treatment or wound healing.

## 1. Introduction

Natural minerals represent an abundant and cost-effective resource, having specific physico-chemical properties which can be utilized in agriculture, ecology, industry, and biomedicine. Among this large group of minerals, some—mainly phyllosilicates but also certain tectosilicates—demonstrate remarkable capacities in terms of interacting with ions, organic substances, and even organisms [1,2,3,4]. Within the tectosilicates, zeolite minerals possess a 3D network of linked SiO_4_ and AlO_4_ tetrahedra with open channels and an overall net negative charge, making them useful as molecular sieves and for cation exchange applications [5]. In addition, zeolites can bind larger organic substances onto their surface [6,7,8]. Due to these particular properties, natural zeolites have been historically exploited for various purposes such as soil amendment, (waste)water treatment, and building materials, as well as in numerous industrial processes and medical and biological applications [9,10].

Larger deposits of clinoptilolite-tuff are generally related to volcanic ash fall and the subsequent deposition and interaction of the tuff with saline water, triggering the devitrification of volcanic glasses into zeolite minerals [11,12]. Depending on the prevalent pressure/temperature conditions, the chemical composition of the volcanic ash, and the aqueous environment, different endmembers of the zeolite family can be formed.

Clinoptilolite-tuff has been found to effectively capture and remove a wide range of substances, both in vitro and in vivo, including microbial toxins [6,13,14], drugs [15,16,17,18], biochemicals [7,19,20,21], and viruses [22,23]. The natural clinoptilolite-tuff used in this work is sourced from a mine located in Nižný Hrabovec (Slovak Republic) [11,12]. This material is characterized by its high clinoptilolite content, a high ion exchange capacity, and very low contents in terms of naturally bound heavy metals [11,12]. To offer an absolutely safe version of the tuff, in particular for prolonged biomedical applications, a rigorous, quality-controlled and patented purification process was performed to reduce the heavy metal contents to innocuous levels [24]. Using the resulting purified clinoptilolite-tuff (PCT), we demonstrated the effective binding of bacterial toxins [25] and allergens [26], and even the inactivation of SARS-CoV-2 in vitro [27].

Despite this success, the overall virucidal potential of PCT remains poorly understood. In this study, we tested a broad range of structurally diverse viruses to tackle this lack of knowledge and to better understand the mechanisms of the binding and inactivation of viruses to PCT. The spectrum of viruses that can be neutralized by PCT was examined using RNA and DNA viruses, encompassing both enveloped and non-enveloped viral types: Human Influenza A (HIA-H1N1) for RNA-enveloped viruses, Human Herpes Simplex virus 1 F (HSV-1F) for DNA-enveloped viruses, and Human Rhinovirus 7 (RV-A7) for RNA non-enveloped viruses. We even expanded our observations to other viral hosts, performing additional testing with animal and bacterial viruses that are non-infectious to humans: Canine Parvovirus 2 (CPV-2) for animal viruses (DNA non-enveloped), MS2 and T4 bacteriophages (RNA and DNA, respectively), and 2 non-enveloped viruses infecting bacteria (Table 1).

## 2. Materials and Methods

### 2.1. Purified Clinoptilolite-Tuff

Purified clinoptilolite-tuff (PCT) was prepared from a high-grade raw material with a low heavy metal content, sourced from an open-pit mine in Nižný Hrabovec (eastern Slovak Republic) [11,12]. The patented purification process, operating based on the ion exchange mechanisms of the clinoptilolite mineral, micronization, and terminal heating, results in the removal of natural impurities and a homogeneous, very-fine-grained particle size [24]. The production process is thoroughly quality assured.

The material was characterized using X-ray diffraction (XRD, D-8 Advance, Bruker, USA) and laser diffraction (Mastersizer 2000, Malvern Panalytical, Kassel, Germany) in order to reveal its mineral composition and particle size distribution. A thorough overview discussing the material characterization (chemical and physico-chemical properties) is presented in the Supplementary Table S1.

In short, PCT has a mean particle size (d0.5) of 3.2 ± 0.02 µm, as determined via laser diffraction. Data from XRD revealed that the powder is predominantly composed of the natural zeolite mineral clinoptilolite. The other minerals found in the tuff include cristobalite, feldspars, and traces of biotite and quartz.

Before use in cell culture, PCT was sterilized with 70% ethanol. The alcohol was removed by centrifugation at 2000× *g* for 10 min and PCT powder was dried for 15 min at room temperature (RT) under the laminar flow. Thereafter the material was suspended in a serum-free medium and diluted to achieve the respective concentrations.

### 2.2. Cell Culture, Virus Propagation and Reagents

The following viruses and their corresponding host cells were obtained from the American Type Culture Collection (ATCC, Manassas, Virginia, USA): Influenza A H1N1 (HIA-H1N1, VR-1469) and MDCK cells (CCL-34), Human Herpes Simplex virus 1 (HSV-1, VR-733) and HEp-2 cells (CCL-23), Human Rhinovirus A-7 (RV-A7, VR-1601) and MRC-5 cells (CCL-171), and Canine Parvovirus (CPV-2, VR-2017) and A-72 cells (CRL-1542). Vero cells were acquired from AddexBio, San Diego, CA, USA (P0014002).

A-72 cells were grown at 37 °C in a DMEM medium (PAN-Biotech, Aidenbach, Germany), supplemented with 5% fetal bovine serum, (FBS, Catus Biotech, Tutzing, Germany), 100 U/mL penicillin, 100 µg/mL streptomycin (AppliChem, Darmstadt, Germany), 2 mM L-Glutamine (Biomedica, Vienna, Austria), 1 mM sodium pyruvate (PAN-Biotech, Aidenbach, Germany), and 1% non-essential amino acids (PAN-Biotech, Aidenbach, Germany). Madin–Darby Canine Kidney (MDCK) cells were maintained in a DMEM medium (PAN-Biotech, Aidenbach, Germany) supplemented with 10% FBS and 2 mM L-Glutamine. HEp-2, Vero, and MRC-5 cells were maintained in EMEM (PAN-Biotech, Aidenbach, Germany), supplemented with 10% FBS and 2 mM L-Glutamine. TPCK-treated Trypsin was purchased from Merck (Vienna, Austria). All cells were handled according to the ATCC’s guidelines.

For infection with HSV-1, a 199 V medium was freshly prepared before use. To prepare this medium, an HBSS solution (Merck-Millipore, Vienna, Austria) was supplemented with 2 mM L-Glutamine and 0.1% sodium bicarbonate (Merck-Millipore, Vienna, Austria), buffered at pH 7.2, and sterile-filtered.

The primary antibodies used for virus labeling were as follows: Influenza A virus HA (C102), Santa Cruz (sc-52025) for Influenza A, HSV-1 gD (DL6)-AlexaFluor 488, Santa Cruz (sc-21719) for Herpes Simplex, ds RNA clone (J2), Szabo-Scandic for Rhinovirus and CPV1-2A1, and Santa Cruz (sc-57961) for Canine Parvovirus.

### 2.3. Amplification of Influenza A H1N1

A highly concentrated virus suspension was obtained via the amplification of the virus in a MDCK cell culture. MDCK cells were seeded at 80–90% confluency in a 75 cm^2^ flask (TPP, Trasadingen, Switzerland). The following day, they were infected with 5 µL of the original ATCC virus suspension (concentration 6.7 × 10^6^ virions per ml), which was diluted in 2 mL of serum-free Eagle MEM medium supplemented with 1 µg/mL TCPK-treated trypsin. After 1 h at 35 °C, 25 mL of medium was added, and the cells were further maintained at 35 °C. After 4 days, the supernatant was collected and cleared via a 5 min centrifugation at 4000× *g*, aliquoted, and stored at −80 °C until further use. The titer of the stock suspension was 55 million infectious virions per ml. For virus quantification, MDCK cells were replaced by Vero cells.

### 2.4. Amplification of Bacteriophages T4 and MS2

Bacteriophages T4 (D4505, DSMZ, Leibniz-Institut, Deutsche Sammlung von Mikroorganismen und Zellkulturen, Griesheim, Germany) and MS2 (DSM 13767, DSMZ, Griesheim, Germany) were propagated according to the DSMZ guidelines in their respective bacterial hosts, *E. coli* strain B, B Luria (DSM 613, DSMZ, Griesheim, Germany), and *E. coli* strain Lederberg W1485 (DSM 5695, DSMZ, Griesheim, Germany).

For the amplification of T4 bacteriophage, M9 complete medium was freshly prepared before use by supplementing an M9 salt solution with 0.5% L-glucose, 1% casoamino acids, 1.5 mM vitamin B1, 2 mM magnesium sulfate and 2.5 mM tryptophan, all from Merck-Millipore (Vienna, Austria). The MS2 bacteriophage was amplified using NZCYM Broth (Carl Roth, Karlsruhe, Germany).

A stock suspension of bacteriophages was prepared by inoculating an exponentially growing *E. coli* culture with the original bacteriophage suspension and incubating it for 24 h at 37 °C under conditions of agitation. After pelleting the bacteria and debris at 4700× *g* for 7 min, the supernatant was cleared with a 0.45 µm membrane filter and the bacteriophage concentration in the filtrate was quantified using a plaque-forming assay.

### 2.5. Adsorption of Human and Animal Viruses onto PCT

The adsorption of viruses onto PCT was carried out by incubating the respective virus suspension in the respective infection media (IM) with PCT in a final volume of 1 mL at the indicated concentrations for 1 h at RT (Table 2). After pelleting PCT via centrifugation for 10 min at 4000× *g*, the supernatant was collected, and its titer was determined using a focus-forming assay, whereas the pellet was used for a desorption assay.

### 2.6. Desorption Assay

To desorb the viruses that were potentially bound to the PCT pellet, the pellet obtained from the condition with the highest PCT concentration was used. The pelleted powder was incubated for 1 h at RT with the respective infection medium under conditions of constant agitation and centrifuged again for 10 min at 4000× *g*. The infectious virus titer in the supernatant was quantified using a focus-forming assay.

The results are expressed as a percentage of the infectious virus released from the powder compared to the virus titer released from a control incubated without PCT. The values obtained from the 4 experiments were expressed as mean ± standard deviation.

### 2.7. Adsorption and Inactivation of Bacteriophages onto PCT

T4 and MS2 bacteriophages were serially diluted in their respective growth media and mixed with 1 mg/mL PCT for 1 h at 37 °C. In the negative controls, PCT was replaced by peptone water (Merck-Millipore, Vienna, Austria). Upon incubation, the samples were centrifuged for 30 min at 4000× *g* in order to pellet the PCT.

The quantification of infectious bacteriophages in the resulting supernatants was performed in duplicate using a plaque-forming assay, as described below.

### 2.8. Quantification of Human and Animal Infectious Virus Particles by Focus-Forming Assay

For each virus, an appropriate focus-forming assay was developed to quantify the infectious virus titer. The specific parameters used for each virus/cell pair are presented in Table 2.

First, permissive cells were seeded onto 24-well plates and, when they reached 80–90% confluency, they were infected with 250 µL of virus-containing supernatant, diluted in its respective infection medium. After one hour of infection at the respective temperature, the virus suspension was replaced with 500 µL of overlay medium, consisting of 1–2% methylcellulose in a medium form. After 30 min at RT to allow the overlay to gel, 1 mL of incubation medium was added on top of the overlay and the plates were further incubated at the respective temperatures for the time necessary to obtain plaques of adequate size.

Due to the absence of cell lysis, the visualization of the infection plaques obtained with human and animal viruses required immunostaining. The common protocol used involved the fixation of the cell layer for 20 min at RT with a fixative solution made of 4% formaldehyde (Science Services, München, Germany) in PBS (PAN-Biotech, Aidenbach, Germany), followed by permeabilization for 10 min at RT with 0.1% Triton X-100 (VWR, Darmstadt, Germany) in PBS. After blocking for 1 h at RT with 5% normal goat serum (NGS, Merck Millipore, Vienna, Austria) in PBS, samples were stained with a primary antibody, which was diluted 200-fold to 400-fold, in a buffer made of 0.01% Triton X-100 and 1% NGS in PBS for 1 h at 37 °C.

After 3 washes with a buffered solution made of 0.01% Triton X-100 in PBS, labeling was performed by incubating the samples for 1 h at 37 °C with a 400-fold dilution of a goat anti-mouse antibody conjugated with CoraLite 488 (ProteinTech, Manchester, UK). After another 3 washes with the above-mentioned washing buffer, the samples were observed via fluorescence microscopy using an Olympus IX83 inverted microscope (Olympus, Hamburg, Germany). Negative controls that lacked the primary antibody were prepared to define the background signal. Each condition examined within the experiment was tested in duplicate or triplicate and each experiment was repeated 3 times. The results were expressed as plaque-forming units (PFU) per ml and represent the mean and standard deviation values of all 3 experiments.

For the labeling of HSV-1, the primary antibody was directly conjugated with Alexa-488 (Thermo Fisher Scientific, Vienna, Austria) and therefore no secondary antibody was used.

### 2.9. Quantification of Infectious Bacteriophages via Plaque-Forming Assay

In order to quantify the phage suspensions, they were first diluted 10-fold in their respective medium and 1 mL of this dilution was mixed with 2 mL of their respective host cells in the growing phase, as indicated in Table 2. After a 30 min incubation at 37 °C, 100 µL of the sample was mixed with 6 mL of the overlay solution, consisting of 0.6% agar–agar (Merck-Millipore, Vienna, Austria), in the respective medium and the mixture was plated on an agar petri dish prepared with the respective agar medium. The petri dish was incubated for 24 h at 37 °C before the lysis plaques could be quantified via direct visualization. The values obtained from the 3 experiments, performed in duplicate, are expressed as the mean ± standard deviation.

## 3. Results

For the standardization and comparison of all experiments, incubations of PCT with the different viruses were all performed for 1 h at RT.

### 3.1. Human Influenza A (HIA-H1N1)

**Figure 1:**
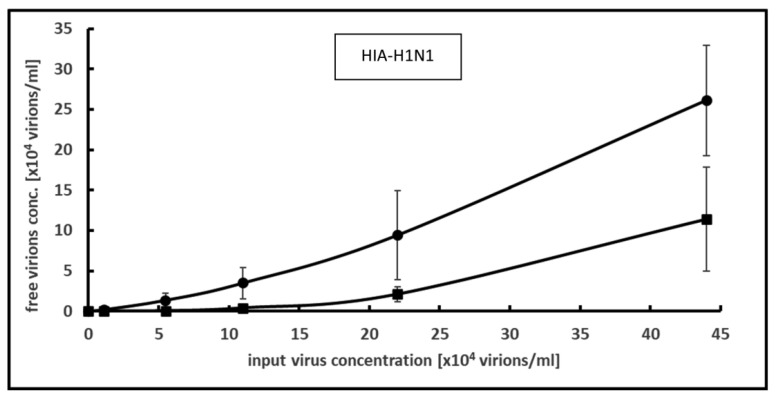
Binding and inactivation of PCT on Human Influenza A (HIA-H1N1) as measured by focus-forming assay. Increasing input virus concentrations were incubated with PCT suspension at 0.2 mg per ml for 1 h at room temperature and free infectious virion concentration was assessed using focus-forming assay. Means and S.D. are shown from 3 independent experiments. (●) without PCT, (■) with PCT.

A constant concentration of 0.2 mg/mL PCT was incubated with increasing concentrations of virus, ranging from 5500 to 440,000 virions per ml. Compared with the control samples without PCT, the addition of PCT consistently led to a decrease in the number of free infectious viral particles (Figure 1). For the highest virus input concentration, this decrease reached up to 56.2%.

Although no plateau, and therefore no saturation, could be achieved, the calculated maximal binding capacity reached 735,000 infectious viral particles per mg PCT.

To determine whether the binding and inactivation of HIA-H1N1 through PCT were reversible, PCT loaded, with viral particles, was subsequently incubated with a culture medium. The infectious virions potentially released from the powder were then quantified in the supernatant.

The desorption rate amounted to 0.022 ± 0.013% of the bound and inactivated virions.

### 3.2. Human Herpes Simplex Virus-1 F (HSV-1F)

After demonstrating that PCT can bind and inactivate Influenza A, taken as a model for RNA-enveloped viruses, we hypothesized that the same may hold true for DNA-enveloped viruses. Therefore, we tested the binding and inactivation of Herpes Simplex virus as a model for this virus family.

The incubation of up to 400,000 virions per ml HSV-1F with 0.2 mg/mL PCT resulted in all infectious virions being bound and inactivated; therefore, no binding capacity could be calculated. By reducing the PCT concentration to 0.02 mg/mL, it was possible to count the infectious virions remaining in the supernatant.

**Figure 2:**
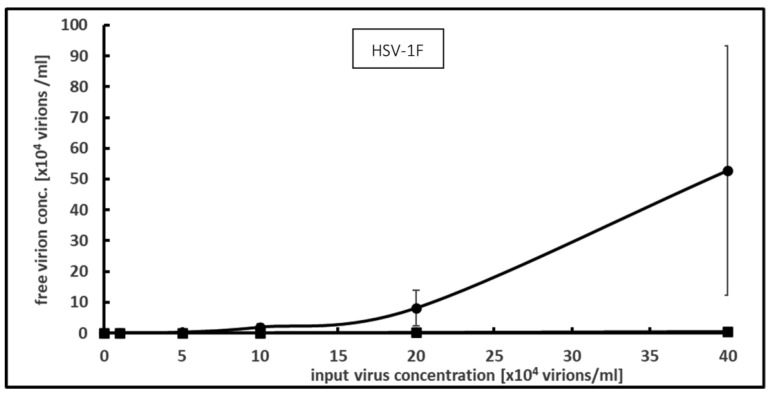
Effect of PCT on HSV-1F as measured by focus-forming assay. Increasing input virus concentrations were incubated with a PCT suspension at 0.02 mg per ml for 1 h at room temperature and the free virus concentration was assessed by focus-forming assay. Means and S.D. values from 3 independent experiments are shown. (●) without PCT, (■) with PCT.

For the highest input virus concentration tested (400,000 virions per ml), a 98.9% drop was registered (Figure 2). Again, no saturation could be attained. The maximal binding capacity was 26.1 million infectious viral particles per mg PCT. For this condition, a desorption rate of 1.2 ± 0.5% was measured.

### 3.3. Human Rhinovirus 7 (RV-A7)

**Figure 3:**
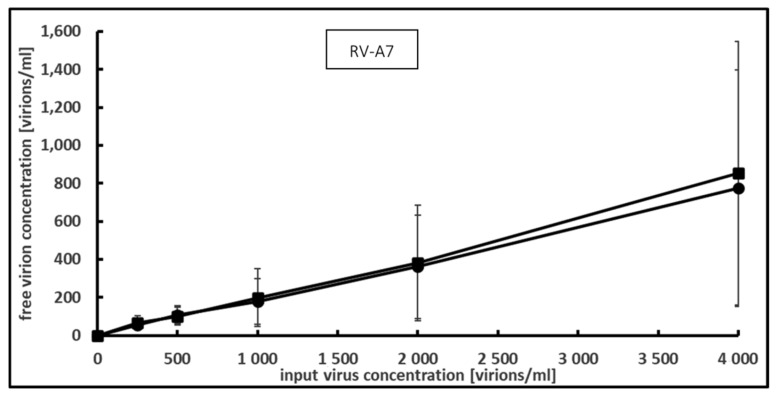
Effect of PCT on RV-A7 as measured by focus-forming assay. Increasing concentrations of virus were incubated with PCT suspension at 0.2 mg per ml for 1 h at room temperature and free virus concentration was assessed by focus-forming assay. Means and S.D. values from 3 independent experiments are shown. (●) without PCT, (■) with PCT.

Since PCT can bind and inactivate HIA-H1N1 and HSV-1F, taken, respectively, as models for RNA- and DNA-enveloped viruses, the question arose if this is still true for non-enveloped human viruses. To test this hypothesis, RV-A7 was chosen as the model for non-enveloped RNA viruses. Increasing virus concentrations were incubated with 0.2 mg/mL PCT. Due to the difficulty of obtaining high concentrations of the viral suspension inherent to this virus, a concentration curve ranging from 250 to 4000 virions per ml was used. Under these conditions, the addition of PCT had no effect on the concentration of infectious virions (Figure 3).

Since no binding occurred, no binding capacity and no desorption rate could be calculated.

### 3.4. Canine Parvovirus-2 (CPV-2)

In order to seek confirmation of the inefficacy of PCT in binding and inactivating Rhinovirus, taken as a model for non-enveloped viruses and, concurrently, to extend our investigation to other hosts than humans, Canine Parvovirus, a member of DNA non-enveloped viruses infecting dogs, was chosen next for examination.

**Figure 4:**
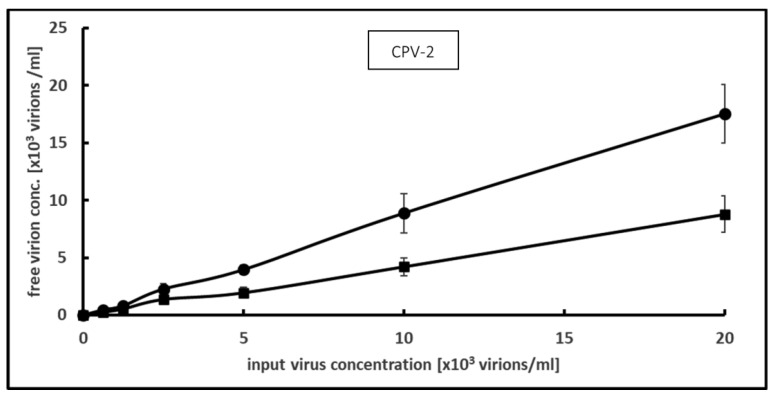
Effect of PCT on CPV-2 as measured by focus-forming assay. Increasing concentrations of virus were incubated with PCT suspension at 0.2 mg per ml for 1 h at room temperature and free virus concentration was assessed via focus-forming assay. Means and S.D. values from 3 independent experiments are shown. (●) without PCT, (■) with PCT.

Testing the binding and inactivation of CPV-2 with 0.2 mg/mL PCT and an input virus concentration ranging from 625 to 20,000, our results revealed that PCT caused a 50% decrease in the infectious viral particle concentration in the supernatant (Figure 4). The maximum interaction capacity and desorption rate were calculated for the highest virus input concentration, at 43,767 viral particles per mg PCT and 5.9 ± 1.6%, respectively.

### 3.5. Bacteriophages T4 and MS2

Observing no binding for RV-A7 and only a modest binding for CPV-2, both non-enveloped viruses, our investigation was extended to non-enveloped bacterial viruses, namely, the bacteriophages T4 and MS2.

Unlike with the previously tested viruses, high yields of bacteriophages can be obtained easily. Accordingly, the results are expressed as log_10_ to improve readability.

The incubation of the bacteriophages with up to 1 mg/mL PCT resulted in no change in the number of infectious viral particles (Table 3). Due to the absence of absorption, no desorption experiment could be performed.

## 4. Discussion

The use of natural minerals or mineral assemblages for biomedical purposes, such as developing antimicrobials, is a practice that dates back to ancient times. Found abundantly in nature, they have been applied in various forms to prevent and treat infections [9,28]. Today, with the rise of antibiotic resistance, the non-specific antimicrobial properties of certain minerals are being revisited due to high levels of interest.

The binding and inactivation of diverse virus strains to, and their inactivation by, phyllosilicates (e.g., clays) and tectosilicates (e.g., zeolites) has frequently been demonstrated [1,2,3,4,22,23,27,29]. Recently, we were able to demonstrate the rapid and effective binding and inactivation of coronaviruses in vitro by purified clinoptilolite-tuff [27], a safe natural zeolite-bearing material characterized by remarkable ion exchange and absorptive properties, after they were extensively purified through a fully quality-controlled process [24].

Building on the insights of that research, we aimed to further explore the virucidal potential of PCT in vitro across a broad spectrum of viruses, including those affecting humans, animals, and bacteria. This investigation encompassed RNA and DNA viruses, as well as both enveloped and non-enveloped species. PCT was introduced to pure viral suspensions and the unbound virions were quantified by plaque and focus-forming assays. Additionally, the possible release of infectious virions from virus-loaded PCT was also quantified using the same methods.

Our findings reveal that the effectiveness of PCT in binding and inactivating different viruses varies significantly. The best antiviral performance was obtained with the Herpes Simplex virus, with nearly 99% of the virus being inactivated by PCT. For Influenza A and Canine Parvovirus, decreased but still significant inactivation rates of 56% and 50%, respectively, were found. By contrast, PCT did not demonstrate any binding or inactivation effects on Rhinovirus, a human non-enveloped RNA virus, and on two bacteriophages differing by their morphologies and genomes.

To investigate the potential of bound viruses to remain infectious and be released from PCT, virus-loaded PCT powder was first incubated with infection medium and infectious virions present in the powder-free supernatant were quantified via a focus-forming assay. For those viruses for which an antiviral effect was demonstrated, very low desorption rates were measured, ranging from 0.022% to 5.9%, underlining the nearly irreversible character of virus binding and inactivation by PCT.

In a previous publication, we reported that PCT could neutralize as many as 40 million coronavirus particles per mg [27]. In the present work, a similar neutralization capacity was found for Herpes Simplex (26 million per mg), whereas a more modest binding capacity was calculated for Influenza A (735,000 per mg). For Parvovirus, the virucidal capacity was further reduced to less than 45,000 virions per mg.

Interestingly, Lipson et al. previously demonstrated the binding of Reovirus, a non-enveloped RNA virus, to kaolinite and montmorillonite clays [30]. This contradicts the results in present studies into PCT and the use of Rhinovirus as a model for human non-enveloped RNA viruses. This observed discrepancy may be attributed to the differences in the methods or to the nature of the viruses or sorbents involved. Of note, the above-mentioned authors highlighted the importance of considering the presence, nature, and concentration of proteins in the binding buffer when evaluating virus binding to minerals. Additionally, the fact that PCT consists of a natural zeolite mineral, whereas bentonite and montmorillonite are clay mineral mixtures, may also account for the observed discrepancy. Although the two types of minerals share a similar crystal structure, being made of interlinked Si-tetrahedrons with some silicon atoms substituted by aluminum, they possess different framework architectures. Therefore, the charge–density and charge–distribution relationships over the surface are not immediately comparable.

The antiviral potential of a natural but non-purified clinoptilolite was previously tested by Assem et al. against two animal RNA viruses: Foot and Mouth Disease Virus (FMDV) and Ephemeral Fever Virus (EFV). FMDV is classified as an enveloped RNA virus, while EFV belongs to the category of non-enveloped RNA viruses [22]. They observed the significant antiviral activity of clinoptilolite regarding both EFV and FMDV. It is important to note that these authors used concentrations of non-purified clinoptilolite that were higher than those used in the present work. This disparity in concentration levels may account for the lack of antiviral effects observed with PCT and Rhinovirus.

Clark et al. conducted a study using a methodology similar to ours, observing the significant binding of Bovine Rotavirus, another non-enveloped RNA virus, to clays and clinoptilolite [1]. This observation raises an intriguing discrepancy between their results, obtained with Bovine Rotavirus, and our findings with Rhinovirus. The divergence in the results may find its origin in the composition of the media used. Clark et al. performed their binding assay in a serum-free medium, whereas we used a medium supplemented with 10% FBS. As discussed above, this could result in significantly different protein concentrations in the binding media, which can strongly influence the binding mechanisms. In addition, apart from the influence of protein concentrations, other factors may also play a crucial role in the interaction between a mineral and a viral component. For example, local charges or hydrophobic pockets present on the surface of the naked viral capsid may also represent critical components of the binding forces [3]. In this context, it is widely recognized that clay minerals and certain tectosilicates exhibit a net negative surface charge at neutral pH values. This is mainly due to the presence of Si-O-Al and Si-OH groups, which are prone to electrostatic interactions [31]. Accordingly, establishing the Si/Al ratio within the framework is critical in determining the hydrophilic or hydrophobic nature of the material. This ratio can vary due to the natural geological origins of aluminosilicates and on account of processes such as acidic and thermal treatments. Given the net negative surface charge of clay minerals and zeolites, it is reasonable to expect that molecules with net positive charges will be adsorbed. However, most proteins and viruses also have a net negative charge at neutral pH values and, in view of the numerous reports showing the adsorption of proteins and viruses onto clays and zeolites, an axiom has been formulated that takes exchangeable cations into account to create a positively charged layer locally, which can in turn serve as an anchor for biological binding partners with net negative charges [32]. By enriching clinoptilolite-tuff with Ca^2+^ cations during the patented cleaning process to produce PCT, we may even have reinforced this behavior.

Electrostatic interactions are thought to represent the driving forces for the initial binding of proteins [33,34]. Though transient, they may bring other hydrophilic or hydrophobic residues closer to local domains on the mineral surface and create additional bonds [33,35]. In this regard, higher flexibility and dynamicity may play important roles because they increase the probability that, after the initial weak binding, further residues “find” adequate binding sites on the mineral through the deformation or rearrangement of the biological structure. This characteristic may represent the key mechanism which differentiates enveloped from non-enveloped viruses when it comes to adsorption onto mineral surfaces. Non-enveloped viruses possess a rigid viral capsid, and the protein residues present on their surface cannot easily be deformed or adapt to the surface of minerals. By contrast, enveloped viruses interact with their environment through a lipid bilayer originating from the budding of virions through the membrane of the host cell. Consequently, the proteins of viral and cellular origin present in the viral envelope are characterized by a high flexibility and dynamicity [36]. This was demonstrated by Vilker et al., who showed that the binding of the enveloped bacteriophage φ6 onto montmorillonite clay resulted in viral envelope distortion at the interface with the mineral and the subsequent partial disassembly of the virions [2]. This may partly explain why we observed a higher susceptibility of enveloped viruses to PCT’s virucidal potential compared to non-enveloped viruses.

A variety of other factors likely play a role in the adsorption of viruses to PCT. As noted by Syngouna et al., the attachment of viruses onto minerals is also driven by agitation, temperature, ionic strength, pH, and possibly other parameters [29]. The authors conclude that defining the factors involved in these interactions is no trivial matter that can be effectively described by a single model.

Due to the small pore size of clinoptilolites, far below that of a virus, the interaction of viruses with PCT is only conceivable on the surface of the mineral. This is not true for clay minerals, where virus binding can also take place between the structural sheets, as previously demonstrated with montmorillonite [37]. Therefore, care must be taken when comparing studies with clays and zeolites.

The decision to use a low concentration of PCT in this study was based on its exceptionally high binding and inactivation capacity towards the Influenza A and Herpes Simplex viruses. Due to technical constraints limiting the concentration of virions in the viral suspension, employing a higher amount of zeolite would have excessively neutralized any virions present. Therefore, reducing the final concentration of PCT was essential in terms of increasing the proportion of infectious virions in the PCT-free fraction up to a point where they could be counted effectively.

For the same reason, it was not possible to reach a maximal virucidal capacity, as evidenced by the absence of a flattened curve in all binding and inactivation curves. This limitation arose from the difficulty of discerning the impact of adding progressively smaller concentrations of PCT to a highly concentrated virus suspension. Consequently, the difference between samples treated with and without zeolite fell within the natural standard deviation of the data, making it challenging to demonstrate the significant differences arising from the addition of PCT.

In this work, we investigated the direct virucidal activity of PCT, omitting to test PCT extracts. PCT itself is insoluble and, therefore, PCT extracts can only act through the ion exchange of certain free heavy metals present in the crystal lattice and subsequent medium enrichment with these metals, which may affect viruses. However, whereas untreated clinoptilolite-tuff naturally contains a certain amount of exchangeable heavy metals, PCT does not, undergoing an extensive cleaning process. It is worth noting that the PCT concentrations used in this work were particularly low, and therefore it was not possible to significantly modify the composition of the medium itself by ion exchange.

Our investigation elucidating the antiviral potential of PCT extended to two bacteriophages, namely viruses that infect bacteria. These bacteriophages were selected based on their diverse genomes and morphologies: MS2 has an RNA genome enclosed within a typical icosahedral shape, whereas T4 has a DNA genome and displays a more complex shape. The latter is formed by the assembly of an elongated icosahedron followed by a cylindrical “tail”, and terminated by “tactile fibres”, which sense the potential host bacterium. Both bacteriophages are devoid of a lipidic envelope.

Our data show that PCT does not affect the antibacterial activity of the two bacteriophages investigated. This finding is significant in light of the antibacterial properties of clinoptilolite. Indeed, natural and modified clinoptilolites possess antibacterial properties [38,39]. Specifically, the ability of clinoptilolite to reduce the bacterial load on wastewater treatment test beds has been shown repeatedly [40,41]. More hypothetically, the antibacterial activity of clinoptilolite can also be leveraged in the management of chronic wounds, which are often infected [42].

Just like wastewater, chronic wounds usually contain a complex mixture of bacteria, which makes them ideal candidates for bacteriophage therapy [43]. In this context, the absence of an impact on bacteriophage bactericidal activity may potentiate their already proven bactericidal capacity of PCT.

## 5. Conclusions

- PCT exhibits a broad spectrum of virucidal activity, ranging from highly effective to almost inactive depending on the virus species considered.
- Our results suggest that enveloped viruses may display higher susceptibility to PCT’s antiviral character than non-enveloped viruses, possibility because of the increased flexibility and dynamicity of viral envelope-associated proteins.
- The net charges of both mineral surfaces and biological binding partners are unlikely to be enough to predict adsorption behavior.
- PCT does not affect two different bacteriophages, revealing that bacteriophages may represent an oversimplified model for other viruses and suggesting that care should be taken when extrapolating results obtained using bacteriophages to other viruses.

## Figures and Tables

**Table 1:** Main characteristics of the viruses used in this study.

Virus	Strain	Host	Structure	Genome Type	Particle Size (nm)
Influenza A H1N1	A/PR/8/34	Human	enveloped	RNA	80–120
Herpes Simplex 1	F	Human	enveloped	DNA	140–180
Rhinovirus 7	68-Cv11	Human	non-enveloped	RNA	30
Canine Parvovirus	Cornell-780916-80	Dog	non-enveloped	DNA	20
Bacteriophage T4		Bacteria	non-enveloped	DNA	80 × 200
Bacteriophage MS2		Bacteria	non-enveloped	RNA	25

**Table 2:** Experimental conditions used for each virus used in this study.

Virus Name		Cell Line/Bacteria	Infection			Overlay	Post-Infection Incubation		
**Name**	Strain	Name	Infection Medium (IM)	Time (min)	Temp. (°C)	Methylcellulose (MC)	Medium	Time (h)	Temp. (°C)
Influenza A H1N1	A/PR/8/34	Vero	serum-free EMEM + 2 µg/mL TPCK-Trypsin	60	35	2% MC in IM	IM	48	35
Herpes Simplex 1	F	HEp-2	199V	120	37	EMEM + 10% FBS + 2% MC	EMEM + 10% FBS	24	37
Rhinovirus 7	68-Cv11	MRC-5	EMEM + 10% FBS	60	34	EMEM + 1% FBS + 1% MC	EMEM + 1% FBS	48	34
Canine Parvovirus	Cornell-780916-80	A-72	serum-free DMEM	60	37	IM + 1% MC	DMEM + 5% FBS	48	37
Bacteriophage T4		*E. coli* B.B (Luria)	M9	30	37	0.6% Agar in IM	M9	24	37
Bacteriophage MS2		Lederberg W1485	NZCYM	30	37	0.6% Agar in IM	NZCYM	24	37

**Table 3:** Quantification of bacteriophages T4 and MS2 upon incubation with 1 mg/mL PCT (+PCT) or without PCT (−PCT) as assessed by plaque-forming assay. Virus concentrations, expressed as viral particles per ml, are transformed in log10 for clarity. Means and S.D. values from 3 independent experiments are shown.

PHAGE	+PCT		−PCT	
	Mean	SD	Mean	SD
MS2	10.28	0.02	10.28	0.03
T4	8.63	0.04	8.74	0.05

## Data Availability

The data presented in this study are available on request from the corresponding author due to sponsor-related restrictions.

## Supplementary Materials

The following supporting information can be downloaded at: https://www.mdpi.com/article/10.3390/microorganisms12081572/s1, Table S1: composition of PCT as measured by XRD, ICP-MS and laser diffraction.

## References

[1] Clark K.J., Sarr A.B., Grant P.G., Phillips T.D., Woode G.N. (1998). In Vitro Studies on the Use of Clay, Clay Minerals and Charcoal to Adsorb Bovine Rotavirus and Bovine Coronavirus. Vet. Microbiol..

[2] Vilker V.L., Meronek G.C., Butler P.C. (1983). Interactions of Poliovirus with Montmorillonite Clay in Phosphate-Buffered Saline. Environ. Sci. Technol..

[3] Chattopadhyay S., Puls R.W. (1999). Adsorption of Bacteriophages on Clay Minerals. Environ. Sci. Technol..

[4] Chrysikopoulos C.V., Syngouna V.I. (2012). Attachment of Bacteriophages MS2 and ΦX174 onto Kaolinite and Montmorillonite: Extended-DLVO Interactions. Colloids Surf. B Biointerfaces.

[5] Haemmerle M.M., Fendrych J., Matiasek E., Tschegg C. (2021). Adsorption and Release Characteristics of Purified and Non-Purified Clinoptilolite Tuffs towards Health-Relevant Heavy Metals. Crystals.

[6] Bočarov-Stančić A., Adamović M., Salma N., Bodroža-Solarov M., Vučković-Đisalov J., Pantić V. (2011). In Vitro Efficacy of Mycotoxins Adsorption by Natural Mineral Adsorbents. Biotechnol. Anim. Husb..

[7] Markoska R., Stojković R., Filipović M., Jurin M., Špada V., Piltaver I.K., Pavelić K., Marković D., Pavelić S.K. (2023). Study of Zeolite Clinoptilolite D-Glucose Adsorption Properties in Vitro and in Vivo. Chem. Interact..

[8] Selvam T., Schwieger W., Dathe W. (2014). Natural Cuban Zeolites for Medical Use and Their Histamine Binding Capacity. Clay Miner..

[9] Mumpton F. (1999). La Roca Magica: Uses of Natural Zeolites in Agriculture and Industry. Proc. Natl. Acad. Sci. USA.

[10] Pansini M. (1996). Natural Zeolites as Cation Exchangers for Environmental Protection. Miner. Depos..

[11] Tschegg C., Rice A.H.N., Grasemann B., Matiasek E., Kobulej P., Dzivák M., Berger T. (2019). Petrogenesis of a Large-Scale Miocene Zeolite Tuff in the Eastern Slovak Republic: The Nižný Hrabovec Open-Pit Clinoptilolite Mine. Econ. Geol..

[12] Tschegg C., Hou Z., Rice A.H.N., Fendrych J., Matiasek E., Berger T., Grasemann B. (2020). Fault Zone Structures and Strain Localization in Clinoptilolite-Tuff (Nižný Hrabovec, Slovak Republic). J. Struct. Geol..

[13] Katsoulos P.D., Karatzia M.A., Boscos C., Wolf P., Karatzias H. (2016). In-Field Evaluation of Clinoptilolite Feeding Efficacy on the Reduction of Milk Aflatoxin M1 Concentration in Dairy Cattle. J. Anim. Sci. Technol..

[14] Goodarzi M., Modiri D. (2011). The Use Clinoptilolite in Broiler Diet to Decrease of Aflatoxin Effects. International Conference on Asia Agriculture and Animal IPCBEE.

[15] Kukobat R., Škrbić R., Vallejos-Burgos F., Mercadelli E., Gardini D., Silvestroni L., Zanelli C., Esposito L., Stević D., Atlagić S.G. (2023). Enhanced Dissolution of Anticancer Drug Letrozole from Mesoporous Zeolite Clinoptilolite. J. Colloid Interface Sci..

[16] Nippes R.P., Macruz P.D., Molina L.C.A., Scaliante M.H.N.O. (2022). Hydroxychloroquine Adsorption in Aqueous Medium Using Clinoptilolite Zeolite. Water Air Soil Pollut..

[17] Coslop T.F., Nippes R.P., Bergamasco R., Scaliante M.H.N.O. (2022). Evaluation of Diazepam Adsorption in Aqueous Media Using Low-Cost and Natural Zeolite: Equilibrium and Kinetics. Environ. Sci. Pollut. Res. Int..

[18] Tondar M., Parsa M.J., Yousefpour Y., Sharifi A.M., Shetab-Boushehri S.V. (2014). Feasibility of Clinoptilolite Application as a Microporous Carrier for pH-Controlled Oral Delivery of Aspirin. Acta Chim. Slov..

[19] Kristo A.S., Tzanidaki G., Lygeros A., Sikalidis A.K. (2015). Bile Sequestration Potential of an Edible Mineral (Clinoptilolite) under Simulated Digestion of a High-Fat Meal: An in Vitro Investigation. Food Funct..

[20] Li Z., Stockwell C., Niles J., Minegar S., Hong H. (2013). Uptake of Sulfadiazine Sulfonamide from Water by Clinoptilolite. Appl. Environ. Soil Sci..

[21] Akgül M., Savak N.B., Özmak M., Dumanlı A.G., Yürüm Y., Karabakan A. (2008). Adsorption of Bovine Serum Albumin (BSA) on Clinoptilolite. Hacet. J. Biol. Chem..

[22] Assem A.M., Amin M. (2019). Studies on Antiviral Activity of Zeolite Against Foot and Mouth Disease and Ephemeral Fever Viruses. ARC J. Anim. Vet. Sci..

[23] Grce M., Pavelić K. (2005). Antiviral Properties of Clinoptilolite. Microporous Mesoporous Mater..

[24] Glock G. (2012). Method for Removal of Heavy Metals. US Patent.

[25] Ranftler C., Nagl D., Sparer A., Röhrich A., Freissmuth M., El-Kasaby A., Shirazi S.N., Koban F., Tschegg C., Nizet S. (2021). Binding and Neutralization of C. Difficile Toxins A and B by Purified Clinoptilolite-Tuff. PLoS ONE.

[26] Ranftler C., Röhrich A., Sparer A., Tschegg C., Nagl D. (2022). Purified Clinoptilolite-Tuff as an Efficient Sorbent for Gluten Derived from Food. Int. J. Mol. Sci..

[27] Nizet S. (2023). Binding and Inactivation of Human Coronaviruses, Including SARS-CoV-2, onto Purified Clinoptilolite-Tuff. Sci. Rep..

[28] Williams L.B., Haydel S.E., Ferrell R.E. (2009). Bentonite, Bandaids, and Borborygmi. Elements.

[29] Syngouna V.I., Chrysikopoulos C.V. (2010). Interaction between Viruses and Clays in Static and Dynamic Batch Systems. Environ. Sci. Technol..

[30] Lipson S.M., Stotzky G. (1984). Effect of Proteins on Reovirus Adsorption to Clay Minerals. Appl. Environ. Microbiol..

[31] Wang C., Guo H., Leng S., Yu J., Feng K., Cao L., Huang J. (2021). Regulation of Hydrophilicity/Hydrophobicity of Aluminosilicate Zeolites: A Review. Crit. Rev. Solid State Mater. Sci..

[32] Carlson G.F., Woodard F.E., Wentworth D.F., Sproul O.J. (1968). Virus Inactivation on Clay Particles in Natural Waters. J. Water Pollut. Control Fed..

[33] Matsui M., Kiyozumi Y., Mizushina Y., Sakaguchi K., Mizukami F. (2015). Adsorption and Desorption Behavior of Basic Proteins on Zeolites. Sep. Purif. Technol..

[34] Armanious A., Aeppli M., Jacak R., Refardt D., Sigstam T., Kohn T., Sander M. (2016). Viruses at Solid–Water Interfaces: A Systematic Assessment of Interactions Driving Adsorption. Environ. Sci. Technol..

[35] Mathé C., Devineau S., Aude J.-C., Lagniel G., Chédin S., Legros V., Mathon M.-H., Renault J.-P., Pin S., Boulard Y. (2013). Structural Determinants for Protein Adsorption/Non-Adsorption to Silica Surface. PLoS ONE.

[36] Schaap I.A.T., Eghiaian F., Georges A.d., Veigel C. (2012). Effect of Envelope Proteins on the Mechanical Properties of Influenza Virus. J. Biol. Chem..

[37] Block K.A., Trusiak A., Katz A., Gottlieb P., Alimova A., Wei H., Morales J., Rice W.J., Steiner J.C. (2014). Disassembly of the Cystovirus Φ6 Envelope by Montmorillonite Clay. MicrobiologyOpen.

[38] Nikolov A., Dobreva L., Danova S., Miteva-Staleva J., Krumova E., Rashev V., Vilhelmova-Ilieva N. (2023). Natural and Modified Zeolite Clinoptilolite with Antimicrobial Properties: A Review. Acta Microbiol. Bulg..

[39] De la Rosa-Gómez I., Olguín M.T., Alcántara D. (2008). Antibacterial Behavior of Silver-Modified Clinoptilolite–Heulandite Rich Tuff on Coliform Microorganisms from Wastewater in a Column System. J. Environ. Manag..

[40] Shoumkova A. (2011). Zeolites for Water and Wastewater Treatment: An Overview. Research Bulletin of the Australian Institute of High Energetic Materials, Special Issue on Global Fresh Water Shortage.

[41] Kallo D. (2001). Applications of Natural Zeolites in Water and Wastewater Treatment. Rev. Miner. Geochem..

[42] Rondas A. (2016). Prevalence and Assessment of (Infected) Chronic Wounds. Ph.D. Thesis.

[43] Steele A., Stacey H.J., de Soir S., Jones J.D. (2020). The Safety and Efficacy of Phage Therapy for Superficial Bacterial Infections: A Systematic Review. Antibiotics.

