# Supplementary material for: Unveiling the Broad-Spectrum Virucidal Potential of Purified Clinoptilolite-Tuff
Source: Microorganisms. 2024 Aug 1;12(8):1572. doi: 10.3390/microorganisms12081572 (PMC11356225; doi:10.3390/microorganisms12081572)
Supplement: Supplementary file 1 [file microorganisms-12-01572-s001.zip › microorganisms-3103206-supplementary.pdf]

Table S1. composition of PCT as measured by XRD, ICP-MS and laser diffraction.

	**Chemical composition: Oxides [wt%]**
Al_2O_3	12
CaO	5
K_2O	1
MgO	0.3
Na_2O	0.5
SiO_2	66
	**Chemical composition: Elements [µg.g-1]**
Aluminum	64,838 ± 1,724
Silicium	307,780 ± 7,279
Calcium	26,171 ± 336
Nickel	4.1 ± 0.3
Cadmium	<0.05
Cesium	/
Barium	49 ± 1
Thallium	0.18 ± 0.003
Lead	1.6 ±0.1
**Ion exchange capacity [mol.kg-1]**	0.97 ± 0.007
**Particle size [µm]**	3.2 ± 0.02
**Si / Al ratio**	5
